# Causal relationship between diabetes mellitus and lung cancer: a two-sample Mendelian randomization and mediation analysis

**DOI:** 10.3389/fgene.2024.1449881

**Published:** 2024-11-25

**Authors:** Xiaolin Yu, Binfan Fu, Taizhen Sun, Xu Sun

**Affiliations:** Department of Integrated Chinese and Western Medicine, The Affiliated Cancer Hospital of Zhengzhou University and Henan Cancer Hospital, Zhengzhou, China

**Keywords:** lung cancer, diabetes mellitus, mediation, Mendelian randomization, serum metabolites

## Abstract

**Background:**

Diabetes mellitus (DM) is the common comorbidity with lung cancer (LC), and metabolic disorders have been identified as significant contributors to the pathogenesis of both DM and LC. The causality between diabetes mellitus and lung cancer is still controversial. Hence, the causal effects of DM on the risk of LC was systemically investigated, and the mediating role of blood metabolites in this relationship was further explored.

**Methods:**

This study utilized a comprehensive Mendelian randomization (MR) analysis to investigate the association between diabetes mellitus and lung cancer. The inverse variance weighted method was employed as the principle approach. MR Egger and weighted median were complementary calculations for MR assessment. A two-step MR analysis was performed to evaluate the mediating effects of blood metabolites as potential intermediate factors. Simultaneously, sensitivity analyses were performed to confirm the lack of horizontal pleiotropy and heterogeneity.

**Results:**

The two-sample MR analysis illustrated the overall effect of type 1 diabetes mellitus (T1DM) on lung squamous cell carcinoma (LUSC) (OR: 1.040, 95% CI: 1.010–1.072, *p* = 0.009). No causal connection was found between T2DM and the subtypes of lung cancer. Two-step MR identified two candidate mediators partially mediating the total effect of T1DM on LUSC, including glutamine conjugate of C6H10O2 levels (17.22%) and 2-hydroxyoctanoate levels (5.85%).

**Conclusion:**

Our findings supported a potentially causal effect of T1DM against LUSC, and shed light on the importance of metabolites as risk factors in understanding this relationship.

## 1 Introduction

Lung cancer (LC) has emerged as a huge health concern to the lives and health of humans, and it is the leading cause of malignancy-related death (Song et al., 2020). Several risk factors have been reported for the development of LC, such as smoking and aging (Alcaraz et al., 2021). In addition, diabetes mellitus (DM) has been identified as an important risk factor for LC. DM, as a metabolic systemic disease, could cause glucose and fatty acid disorders. DM was the common comorbidity with LC (Danila et al., 2020). The abnormality of the glucose, amino acid, and fatty acid metabolites has been characterized as an important cause in the development of cancer during the pathogenesis of LC (Wang et al., 2020). The disorders in nutrient metabolism could be the intrinsic reason for the incidence of DM and LC.

Diabetes mellitus is one of the most prevalent metabolic diseases worldwide, which contributes considerably to the global disease burden (Farshadpour et al., 2022). With the current changes in lifestyles and environments, the prevalence of DM has been increasing (Tan et al., 2023). DM is classified into two main types, namely, type 1 DM (T1DM) and type 2 DM (T2DM). T1DM is characterized by an autoimmune destruction of insulin-producing cells in the pancreas and subsequently causes an absolute lack of insulin (Adinortey et al., 2022). The central feature of T2DM is insulin resistance with the progressive non-autoimmune loss of β-cell insulin secretion (Qin et al., 2022). The control of glucose is intimately connected with the cancer occurrence. A recent study investigated the association of glucagon-like peptide-1 receptor agonists with cancers and illustrated the evidence of this glucose control drug with cancer risks (Sun et al., 2024).

Mendelian randomization (MR) is used to explore the potential causal effects of exposure on outcomes via genetic variants (Zuber et al., 2020). Compared with conventional clinical research methods, MR is less affected by confounding factors and reverse causation (Chen et al., 2023a). Furthermore, MR can be used to investigate the potential mediators that could influence disease outcomes with exposure factors (Fan et al., 2022). Although a previous study has reported that the risk of lung cancer is genetically predicted with regard to T2DM and fasting insulin concentrations (Pearson-Stuttard et al., 2021), the underlying pathogenesis remains unclear.

Hence, we conducted an MR analysis to systemically investigate the causal effects of DM on the risk of LC, and the underlying mediating role of blood metabolites in this relationship.

## 2 Materials and methods

### 2.1 Study design

**FIGURE 1:**
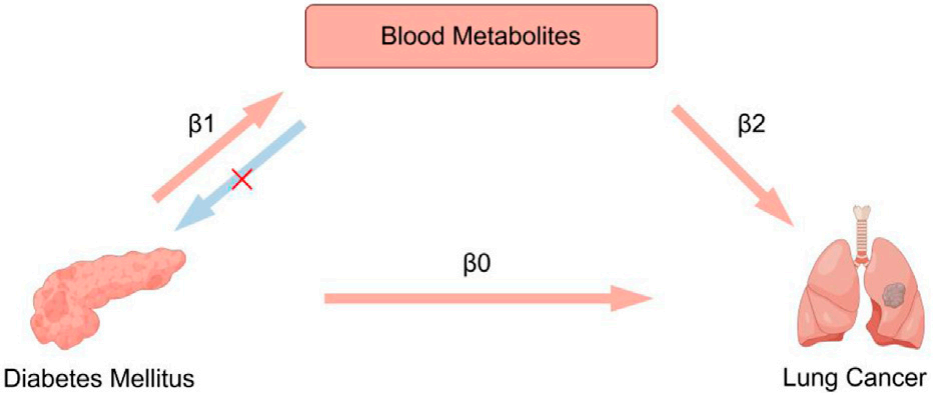
Overview of the study design. A two-step MR analysis was performed to evaluate the mediating effects of blood metabolites as potential intermediate factors. First, the total direct effect of diabetes mellitus on lung cancer risk was calculated by two-sample MR analysis (β0). Second, we estimated the effect of diabetes mellitus on blood metabolites (β1), and the effect of blood metabolites on lung cancer (β2). The indirect effect (β1 × β2) was estimated. Finally, we calculated the proportion of the mediated effect (β1 × β2/β0).

We performed this study using the STROBE-MR guidelines (Supplementary Table S1) (Skrivankova et al., 2021). Genetic variants satisfying the following three core assumptions of an instrumental variable are required for the MR approach (Burgess et al., 2015): 1. Exposures and instrumental variables (IVs) must be significantly related, 2. IVs should not be associated with confounding factors, 3. IVs should affect the outcome solely through exposure and not through other pathways. All data have been approved by the corresponding ethics committee during the enrolling stage, and are publicly available. This study utilized a two-sample Mendelian randomization analysis to investigate the association between diabetes mellitus and lung cancer, as well as to explore the potential mediating effects of blood metabolites on these relationships using a two-step Mendelian randomization analysis. The study design is showed in Figure 1.

### 2.2 Data source

Genome-wide relationship studies (GWAS) data on T2DM were derived from 159,208 participants (2,676 cases and 132,532 controls) (Scott et al., 2017). GWAS data on T1DM were derived from 520,580 participants (18,942 cases and 501,638 controls) (Chiou et al., 2021). For LC, the GWAS data were obtained from FinnGen Consortium (412,181 participants) (Kurki et al., 2023). The genetic effect of the corresponding SNPs on blood metabolites was obtained from 8,299 individuals from the Canadian Longitudinal Study on Aging (CLSA) cohort (Chen et al., 2023b). All individuals included in the study were of European ancestry and the information is shown in Table 1.

**TABLE 1:** Overview of GWAS data used in the MR Analyses.

	GWAD ID/Consortium	Trait	Year	Population	Sample Size	Case	Sex (% Female)	Age (years)	PMID	Author
Exposure	GCST90014023	T1DM	2021	European	520,580	18,942	54.48%	-	34012112	Chiou J
Exposure	GCST004773	T2DM	2017	European	159,208	26,676	55.88%	54.7	28566273	Scott RA
Outcome	FinnGen R10	LUSC	2023	European	412,181	1,510	11.59%	71.3	36653562	Kurki MI
Outcome	FinnGen R10	LUAD	2023	European	412,181	1,590	34.09%	69.5	36653562	Kurki MI
Outcome	FinnGen R10	SCLC	2023	European	412,181	717	18.55%	70.4	36653562	Kurki MI
Mediator	CLSA	Metabolites	2023	European	8,299	8,299	50.9%	62.4	36635386	Chen YH

Abbreviations: LUAD, lung adenocarcinoma; LUSC, lung squamous cell carcinoma; SCLC, small cell lung cancer.

### 2.3 Selection of instrumental variable

Genetic instruments that met the level of *p*-value <5 × 10^−8^ were selected as primary single nucleotide polymorphisms (SNPs). Linkage disequilibrium (LD) was generated due to the physically closely located genetic variances in the genome that tended to be inherited together. The assumption of independence and random assignment was violated by the markers with LD. The genetic instruments were removed the LD regions and the high LD genomic loci were excluded from the selected SNPs to avoid non-random associations (*r* ^2^ < 0.001, kb = 10,000). The F-value statistics reflected the strength of instrumental variables and were calculated to remove the weak instrumental variables and the threshold was set at 10. Finally, we aligned the effect alleles for each SNP allele with the reference panels to ensure the accuracy and consistency of the data. We harmonized the direction of SNPs and removed palindromic sequences and incompatible SNPs.

### 2.4 MR and mediation analysis

Mendelian randomization is an epidemiological approach that assesses the causal inference between the exposure and outcome using instrumental variables from genomic variants. Two-sample MR (TSMR) is applied to evaluate the causal effects of two independent factors. We could perform similar randomizations such as RCT using the TSMR method due to the random segregation of alleles and subsequently compare the effects on outcomes from corresponding groups. We performed the two-sample MR analysis to assess the primary causal relationship between DM and each subtype of LC. The inverse variance weighted (IVW) method was employed as the principle approach because of the robust statistic power when SNPs were valid (Lin et al., 2021). The MR Egger and weighted median were complementary calculations for MR assessment. The MR egger method could estimate the effect for atypical SNPs; however, its statistical power was relatively weak (Burgess and Thompson, 2017). The weighted median method provided robust estimation when valid SNPs were more than 50%, and it was suitable for high pleiotropy (Bowden et al., 2016).

A two-step MR analysis was performed to evaluate the mediating effects of blood metabolites as potential intermediate factors. First, the total direct effect of DM on LC risk was calculated by two-sample MR analysis (β0). Second, we estimated the effect of DM on blood metabolites (β1), and the effect of blood metabolites on LC (β2). The indirect effect (β1 × β2) was estimated. Finally, we calculated the proportion of the mediated effect (β1 × β2/β0).

### 2.5 Sensitive analysis

We conducted the sensitive analyses using TwoSampleMR (version 0.5.6) and MRPRESSO packages (version 1.0) in the R software (version 4.2.2). Cochran’s Q test was used to detect the heterogeneity. The random effect model was selected for high heterogeneity, whereas the fixed effect model was selected for low heterogeneity. We performed the MR Egger regression to estimate the presence of horizontal pleiotropy to eliminate the IVs influencing the outcome from alternative pathways other than the exposure. MR-PRESSO was conducted to exclude outliers and eliminate detected pleiotropy. The leave-one-out method was employed to estimated the robust of the results. The reverse MR was conducted to evaluate the existence of reverse-direction causal association.

## 3 Results

### 3.1 Total effect of DM on lung cancer

**FIGURE 2:**
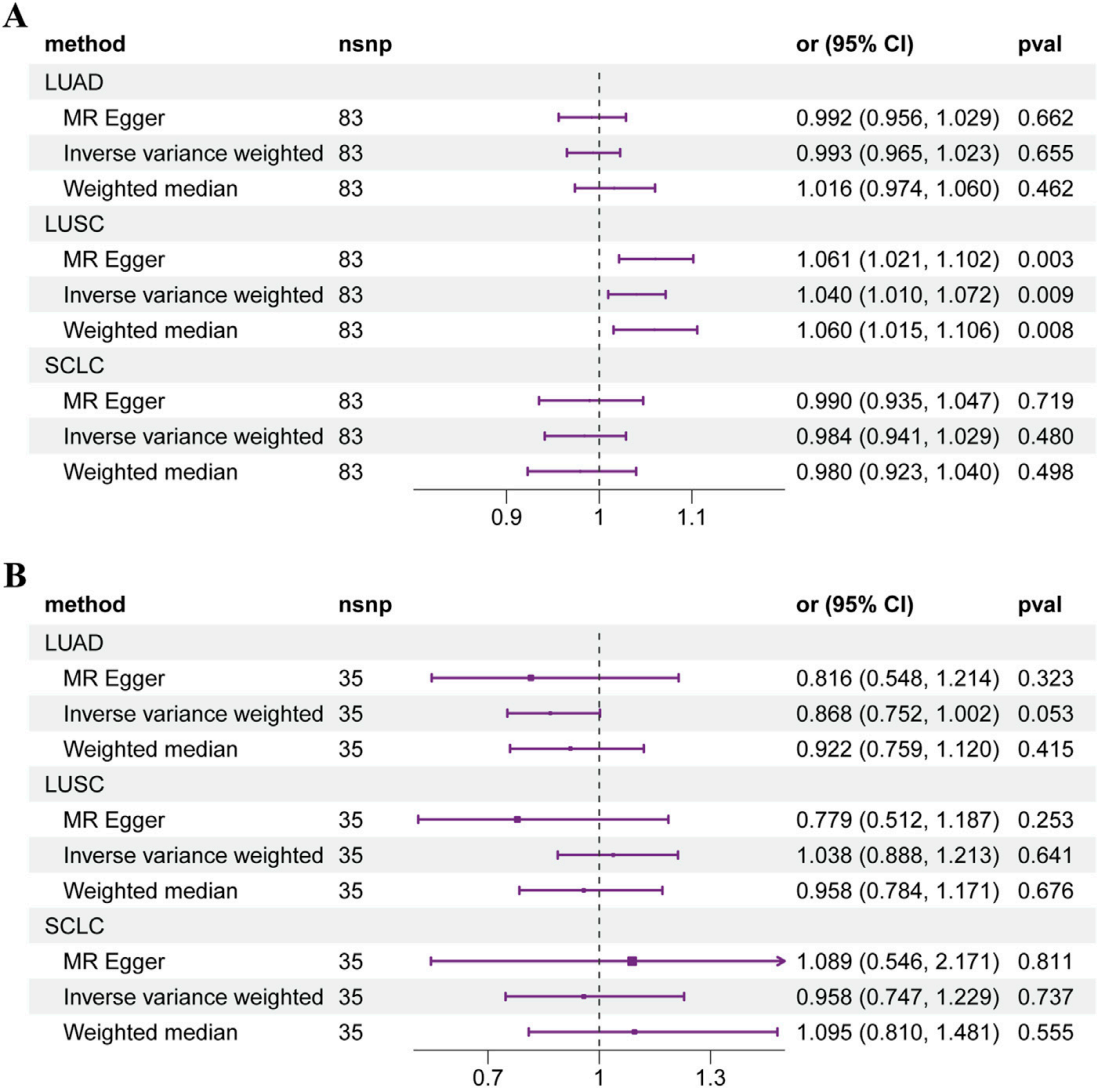
Forest plot of Mendelian randomization results of diabetes mellitus on lung cancer. **(A)** T1DM. **(B)** T2DM. Abbreviations: LUAD: lung adenocarcinoma, LUSC: lung squamous cell carcinoma, SCLC: small cell lung cancer.

As shown in Figure 2A, two-sample MR analyses illustrated the overall effect of T1DM on lung squamous cell carcinoma (LUSC) (OR: 1.040, 95% CI: 1.010–1.072, *p* = 0.009). Each SD increase in genetically predicted T1DM was associated with a 4.0% higher risk of LUSC. The funnel, scatter and leave-one-out plots were shown in Supplementary Figures S1–S3. None of the causal linkages were found in lung adenocarcinoma (LUAD) and small cell lung cancer (SCLC) (Supplementary Table S2). No causal effect of LUSC on T1DM was found for reverse MR (Supplementary Table S3). Three methods of MR displayed adequate consistency. No significant heterogeneity was observed (*p* = 0.505). The MR Egger regression analysis revealed no potential horizontal pleiotropy (*p* = 0.103).

For T2DM, no causal connection was found between T2DM and the subtypes of LC (Figure 2B). The overall effect of two-sample MR showed that genetic predisposition to T2DM is not associated with the risk of LUAD (OR: 0.868, 95% CI: 0.752–1.002, *p* = 0.053), LUSC (OR: 1.038, 95% CI: 0.888–1.213, *p* = 0.641), and SCLC (OR: 0.958, 95% CI: 0.747–1.229, *p* = 0.737). The details of the results were shown in Supplementary Table S2.

### 3.2 Effect of metabolites on lung cancer

The genetically predicted effects of blood metabolites on each subtype of LC are depicted in Supplementary Table S4. A total of 1,400 blood metabolites was used to calculate the causal effect on LC. We screened 873 positive results that met the selecting criteria of *P* (IVW method) < 0.05. The direction of three MR methods was consistent. Significant associations were noted in higher LC for each SD increased in genetically predicted metabolites. The heterogeneity and horizontal pleiotropy analyses are shown in Supplementary Tables S5, S6, respectively.

### 3.3 Effect of DM on metabolites

The effects of genetically predicted T1DM on blood metabolites were estimated by two-sample MR analysis (Supplementary Table S7). Seven metabolites exerted a causal effect on T1DM and met the selecting criteria of *P* (IVW method) < 0.05; the direction of MR methods was consistent (Supplementary Figure S4). Six metabolites exerted a positive causal effect and one negative effect.

### 3.4 Mediating effect of metabolites

As a mediator, the effect of each metabolite is shown in Table 2. Glutamine conjugate of C6H10O2 levels explained 17.22% of the total effect of T1DM on LUSC. The 2-hydroxyoctanoate levels accounted for 5.85% of the total effect. The individual mediated effect of metabolites explained the causal association between T1DM and LC. The results of reverse MR validated causal evidence between the selected mediating metabolites on both LUSC and T1DM (Supplementary Table S8).

**TABLE 2:** Proportion of the effect of type 1 diabetes mellitus on lung squamous cell carcinoma mediated by blood metabolites.

Mediation	Exposure	Outcome	Total effect β0	Direct effect A β1	Direct effect B β2	Mediation effect β	Mediated proportion %
Glutamine conjugate of C6H10O2 levels	T1DM	LUSC	0.039 (0.010–0.069)	0.014 (0.001–0.026)	0.499 (0.121–0.877)	0.007	17.220
2-hydroxyoctanoate levels	T1DM	LUSC	0.039 (0.010–0.069)	0.014 (0.001–0.026)	0.168 (0.019–0.317)	0.002	5.850

Total effect: T1DM, on LUSC, Direct effect A: T1DM, on mediation, Direct effect B: mediation on LUSC.

Abbreviations: T1DM: type 1 diabetes mellitus, LUSC: lung squamous cell carcinoma.

## 4 Discussion

In this MR study, we used a comprehensive assessment of the causal connection between DM on LC. T1DM is significantly correlated with an elevated risk of developing LUSC. Furthermore, a two-step MR analysis revealed that the blood metabolites partially mediated the causal effect of T1DM and LUSC. Our findings shed light on the importance of metabolites as risk factors in understanding the relationship between T1DM and LUSC.

The large metabolic profile data provided high throughput screening and detection in diabetes mellitus (Ottosson et al., 2020). During DM, the contents of different metabolites could cause systemically distinct alterations in blood (Wang et al., 2022a). The risk of diabetic complications and other severe diseases was significantly increased due to the abnormal metabolic changes. Previous research had reported that DM was the risk factor for certain types of cancers, especially lung, pancreatic, breast, and liver cancer (Shahid et al., 2021; Su et al., 2022). Additionally, effective glycemic control treatment has been shown to improve survival rates of post-diagnosis and reduce the risk of developing lung cancer (Duncan and Schmidt, 2009).

Lung cancer constitutes a principal cause of cancer-related mortality globally, underscoring the importance of comprehending its risk factors for effective early screening and prevention strategies. Tobacco smoking represents the predominant risk factor for lung cancer, with empirical evidence indicating a positive correlation between lung cancer incidence and both the duration of smoking and the quantity of daily tobacco consumption (Durawa et al., 2024). Age emerges as a pivotal risk factor, with elderly individuals exhibiting a markedly elevated risk of developing lung cancer (Gallina et al., 2022). Moreover, gender is an independent risk factor, with men demonstrating a greater susceptibility to lung cancer than women. This disparity is likely attributable to differences in smoking habits and environmental exposures (Shrestha et al., 2022). Additionally, factors such as environmental pollution, occupational exposure, and chronic pulmonary diseases are recognized as potential risk factors for lung cancer (Leiter et al., 2023).

The coexistence of diabetes and lung cancer is frequently observed in clinical settings, leading to increased complexity in disease management. Diabetes and lung cancer share several risk factors, including smoking, advanced age, and metabolic syndrome, which contribute to the complex interplay in their pathogenesis (Li et al., 2024a). Diabetes has an impact on various histological types of lung cancer. A study indicated that patients with SCLC who also had diabetes exhibited significantly shorter survival rates compared to those without diabetes (Tas et al., 2024). In the context of LUAD, diabetes notably raised the risk of developing this type of cancer, potentially as a result of metabolic disturbances associated with the diabetic condition. (Chen et al., 2023c). In LUSC, an observational study demonstrated that the severity of diabetes was significantly associated with prognosis (Su et al., 2022). For large cell carcinoma, the current research was relatively limited, but studies on NSCLC indicated possible connections between their risk factors and prognosis. This underscores the need for further clinical investigation in this area.

The pathogenesis of LC has gained widespread attention and therefore, metabolic abnormalities have been emerging as a valuable research direction (Zhong et al., 2023). The rapid growth and proliferation of tumor cells require nutrient acquisition, which has been characterized as an aberrant metabolic mechanism (Wang et al., 2022b). Diabetes mellitus could induce the disorders of glycometabolism and lipid metabolism (Feng et al., 2015). Further metabolomics evidence has demonstrated the increasing glucose and decreasing lactate and phospholipid levels in patients with LC (Louis et al., 2016), and phospholipid composition was the important biomarkers for lung cancer (Marien et al., 2015). Therefore, metabolites possess considerable potential to serve as a conduit for further research on the relationship between diabetes mellitus and lung cancer.

Diabetes mellitus has been reported as an independent risk factor for LC (Lee et al., 2013). The association between T2DM and LC is still controversial (Harding et al., 2015). However, the incidence of LC was significantly elevated for T1DM (Sona et al., 2018), which was consistent with our results. We investigated the genetically predicted causal effects between DM and LC through metabolites. The systemic MR analysis provided evidence of the causal effects on T1DM and LUSC, indicating the increased cancer risk for this patient population. The available data are insufficient for T2DM to confirm the genetically prediction of the causal relationship by MR analysis. However, the presence of insulin resistance could elevate the risk of LC (Argirion et al., 2017). Insulin resistance state enhanced the inflammatory responses and promote the proliferation and invasion of tumor cells (Iyengar et al., 2016). Insulin resistance could result in the abnormal IGF axis; its phosphorylation promotes phosphatidylinositol-3 kinase (PI3K) and mitogen-activated protein kinase (MAPK) pathways and enhances the epithelial–mesenchymal transition (EMT) (Zhan et al., 2024). These findings may offer empirical support for future investigations into the relationship between T2DM and lung cancer.

Our study demonstrated the causal effect of T1DM and LUSC could be mediated by two kinds of serum metabolites. 2-Hydroxyoctanoate is a kind of hydroxy fatty acid. Although no direct experimental evidence exists for the biological effect on T1DM or LUSC, previous studies have reported the potential relationship between them. Hydroxy fatty acids could be linked to other fatty acids by an ester bond, thereby forming a fatty acid complex called fatty acid esters of hydroxy fatty acids (FAHFAs) (Li et al., 2024b). FAHFAs are the newly identified potential biomarkers for DM (Bogojevic et al., 2024), and they also play an important role in tumor progression. The levels of certain FAHFAs are significantly lower in breast cancer patients compared to healthy individuals, suggesting their potential as cancer biomarkers (Qin et al., 2023; Playdon et al., 2017). Additionally, FAHFAs can inhibit the NF-κB signaling pathway, reducing the secretion of pro-inflammatory cytokines and thus altering the tumor immune microenvironment (Li et al., 2024b; Deng et al., 2016). Moreover, FAHFAs have been observed to exert anti-apoptotic effects in colon cancer cells, thereby promoting tumor formation (Rodríguez et al., 2019).

Glutamine-conjugated complexes have been identified as new risk factors for both T1DM and LUSC. As an important participant in amino acid metabolism, abnormal glutamine levels have been associated with the risk of DM (Sriboonvorakul et al., 2021). Glutamate and glutamine metabolism exert a huge burden on the cardiovascular system, insulin sensitivity, and microvascular complications associated with T1DM (Mathew et al., 2019). Glutamine metabolites are one of the main nutrients for cancer survival and growth (Venneti et al., 2015). Targeting glutamine metabolism with glutamine analogs has been proposed for treating cancer (Corchado-Cobos et al., 2022). Altogether, our findings on fatty acids and amino acids supported the linkage between T1DM and LC, and provide epidemiological clues for further exploration. The causal effect of T1DM on LUSC could be mediated by serum metabolites.

To our knowledge, this is the first MR analysis to evaluate the relationship between DM and LC risk and assess the mediating effects of blood metabolites. In all, we provide evidence of the relationship between T1DM and LUSC through genetically predicted effects. T1DM could increase the risk of LUSC, and fatty acid and amino acid metabolites could exert the mediating effect. Reverse MR helps to clarify the direction of causal relationships for more reliable inferences. Our study confirmed that the causal link between T1DM and LUSC is unidirectional: T1DM may raise the risk of LUSC, but LUSC does not influence T1DM. Similarly, reverse MR analysis of blood metabolites also demonstrated the unidirectionality of the mediating effect. The results dismisses reverse causality, strengthening our causal link between T1DM and LUSC, and implies that diabetes-related mechanisms like insulin resistance and metabolic syndrome may significantly influence the risk of LUSC.

This study has certain limitations. First, our study only included the European population, which could influence the generalization of the results to other populations. Second, the TSMR research involved only the analysis of GWAS summary data, further stratification analysis including more covariates, such as age, gender, and stage, could be required. Our findings did not demonstrate a causal relationship between T2DM and lung cancer, potentially due to the presence of confounding factors. Furthermore, despite our efforts to control for potential pleiotropy, completely eliminating such influences in the MR analysis process remains challenging, which may have also impacted the results. Third, the MR analysis indicated the exposure levels of the full life duration and could not reflect the accurate influence of exposure changes.

## 5 Conclusion

Our findings supported a potentially causal effect of T1DM against LUSC, and shed light on the importance of metabolites as risk factors in understanding this relationship.

## Data Availability

The original contributions presented in the study are included in the article/Supplementary Material, further inquiries can be directed to the corresponding author.

## Supplementary material

SUPPLEMENTARY FIGURE S1 Funnel plot of the causal relationships between type 1 diabetes mellitus and lung squamous cell carcinoma using different MR methods.

SUPPLEMENTARY FIGURE S2 Scatter plot of the causal relationships between type 1 diabetes mellitus and lung squamous cell carcinoma using different MR methods.

SUPPLEMENTARY FIGURE S3 Leave-one-out plot of the causal relationships between type 1 diabetes mellitus and lung squamous cell carcinoma using different MR methods.

SUPPLEMENTARY FIGURE S4 Forest plot of Mendelian randomization results of diabetes mellitus on blood metabolites.

## Abbreviations

FAHFAs, fatty acid esters of hydroxy fatty acids; GWAS, genome-wide relationship studies; IVW, inverse variance weighted; LC, lung cancer; LD, linkage disequilibrium; LUAD, lung adenocarcinoma; LUSC, lung squamous cell carcinoma; MR, Mendelian randomization; SCLC, small cell lung cancer; SNPs, gingle nucleotide polymorphisms; T1DM, type 1 diabetes mellitus; T2DM, type 2 diabetes mellitus; TSMR, two-sample Mendelian randomization.
