# Supplementary figures and images for: Causal relationship between diabetes mellitus and lung cancer: a two-sample Mendelian randomization and mediation analysis
Source: Front Genet. 2024 Nov 25;15:1449881. doi: 10.3389/fgene.2024.1449881 (PMC11625780; doi:10.3389/fgene.2024.1449881)

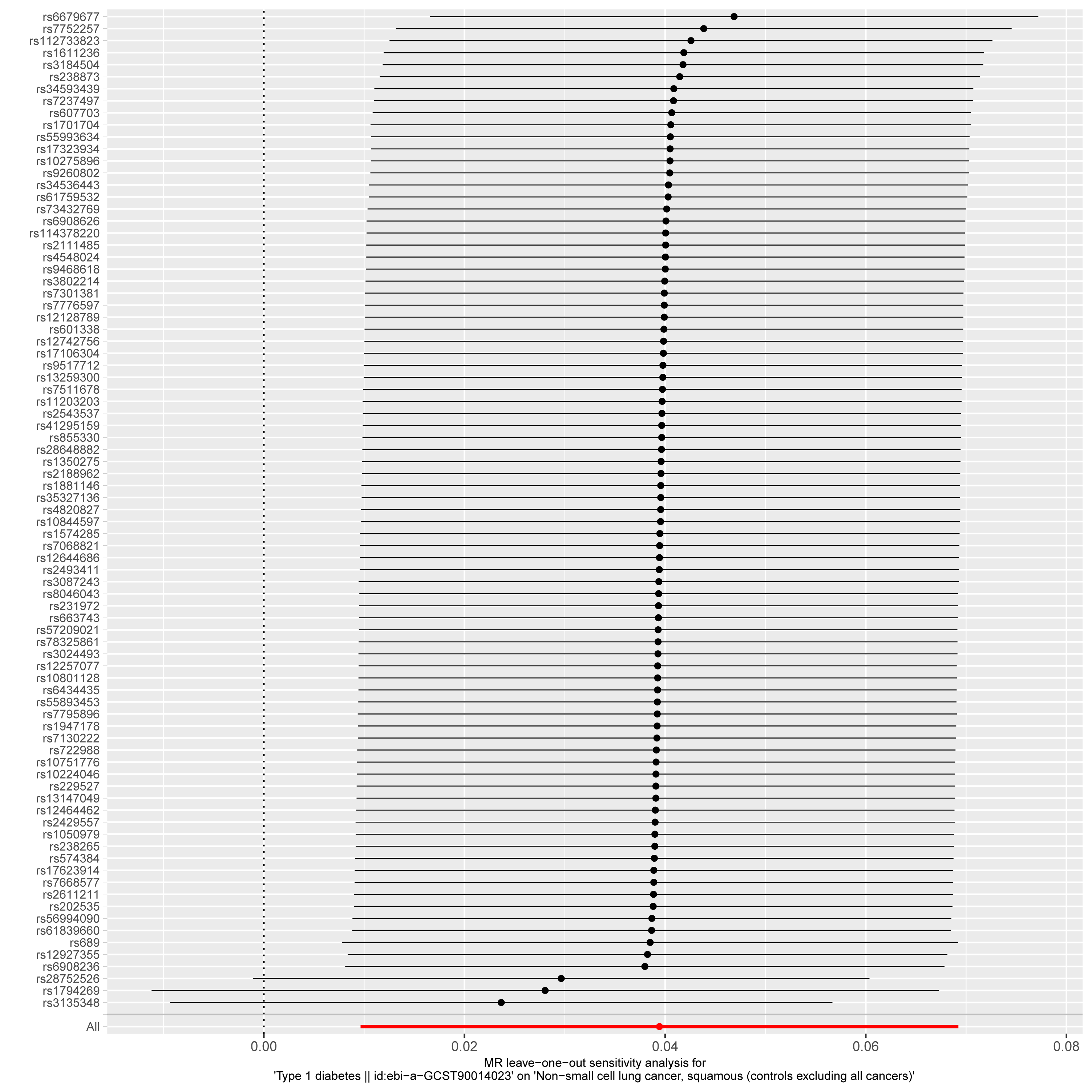

Supplement: Supplementary file 4 [file Image3.tif]

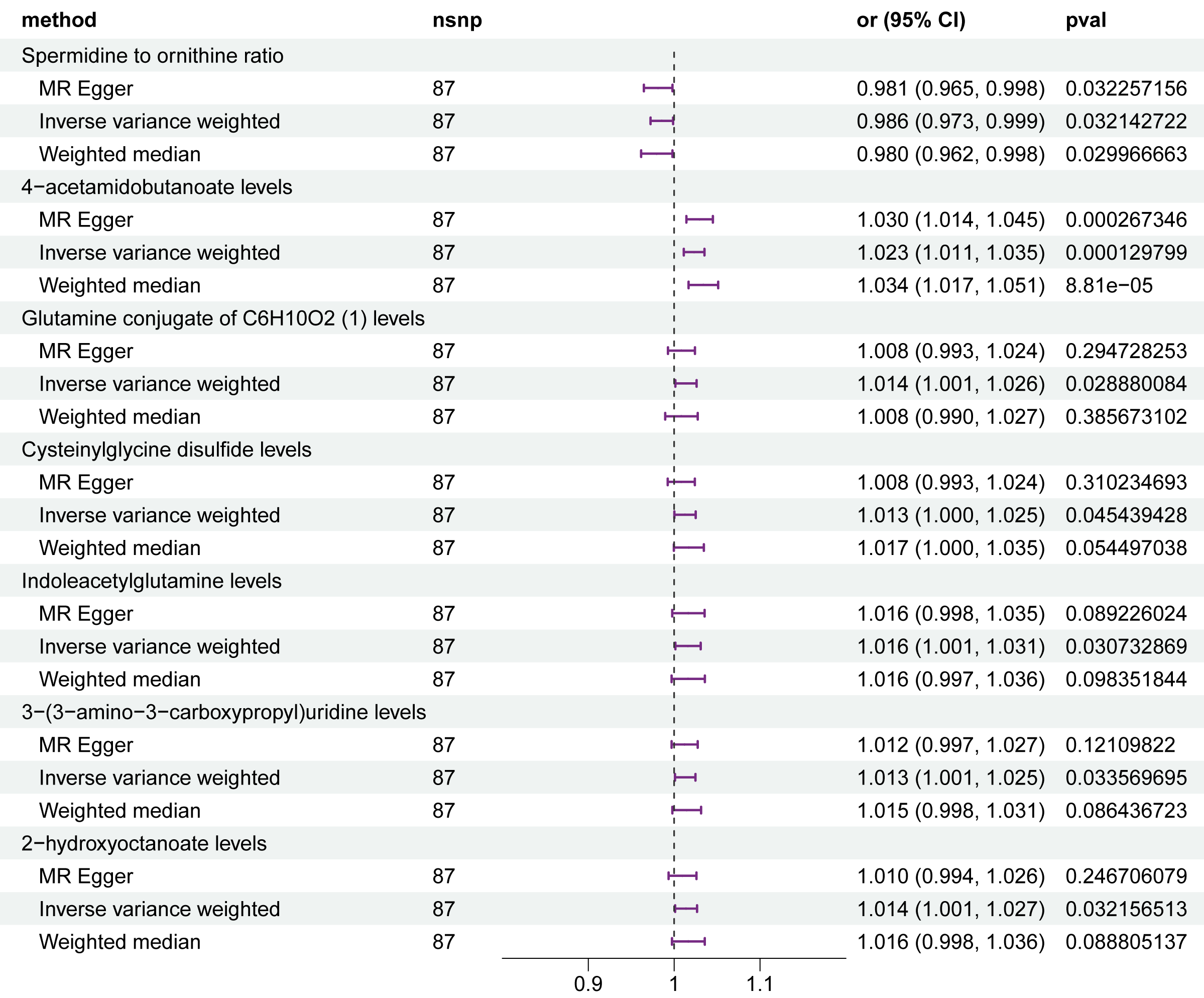

Supplement: Supplementary file 5 [file Image4.tif]

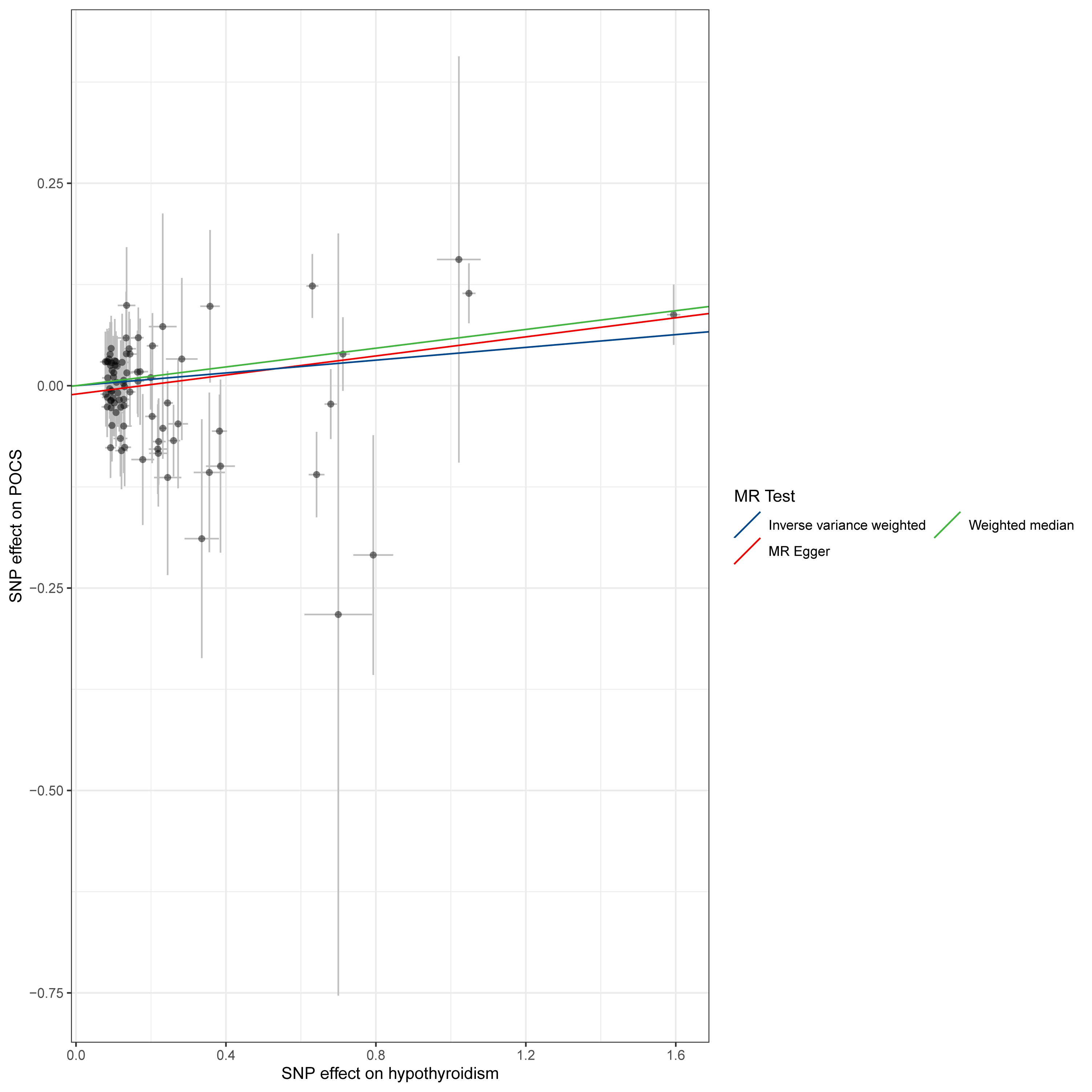

Supplement: Supplementary file 6 [file Image2.tif]

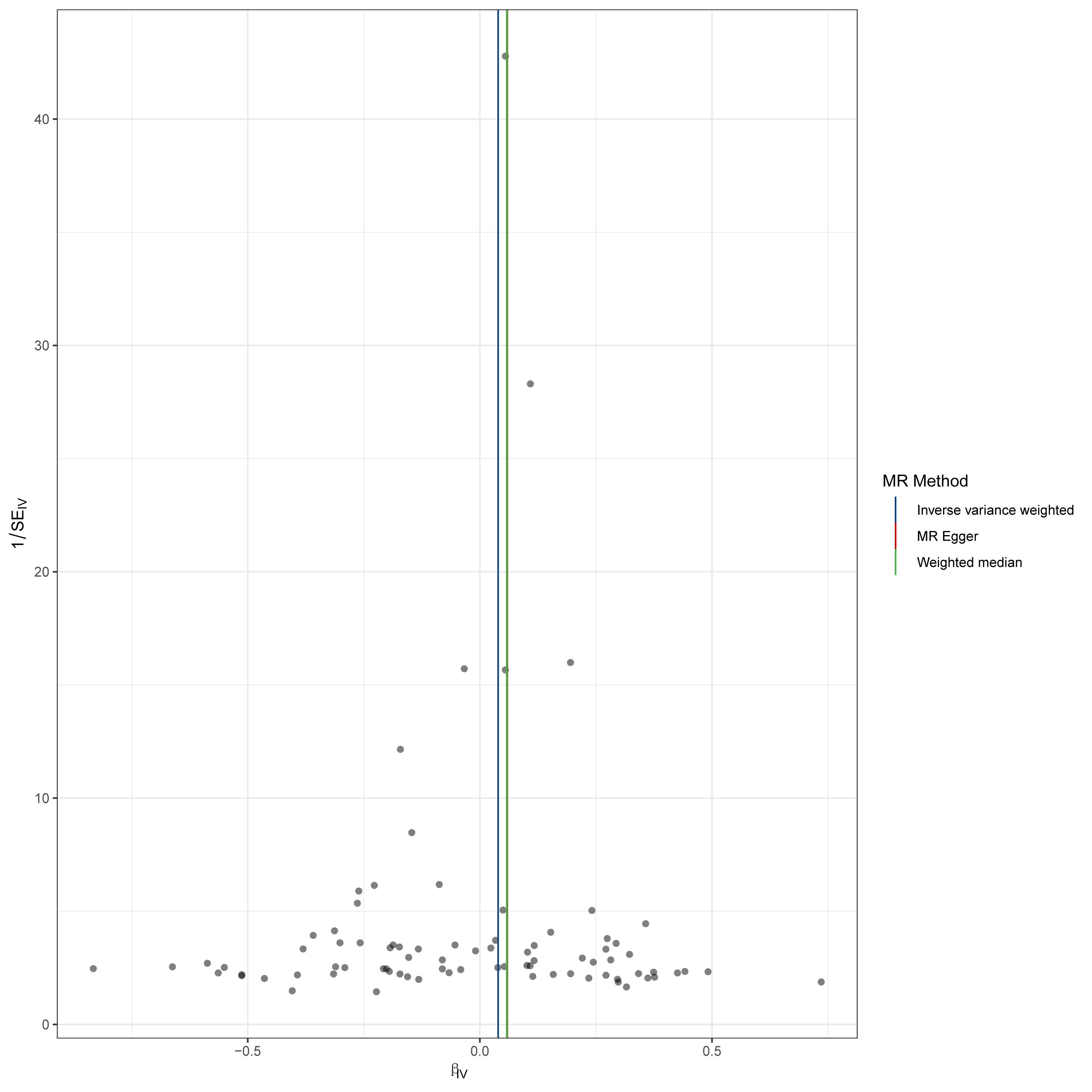

Supplement: Supplementary file 8 [file Image1.tif]
