# Supplementary material for: Causal relationship between diabetes mellitus and lung cancer: a two-sample Mendelian randomization and mediation analysis
Source: Front Genet. 2024 Nov 25;15:1449881. doi: 10.3389/fgene.2024.1449881 (PMC11625780; doi:10.3389/fgene.2024.1449881)
Supplement: Supplementary file 11 [file DataSheet1.docx]

**Table S1 STROBE-MR checklist of recommended items to address in reports of Mendelian randomization studies**^1^ ^2^

| **Item No.** | **Section** | **Checklist item** | **Relevant text from manuscript** |
| --- | --- | --- | --- |
| 1 | **TITLE and ABSTRACT** | Indicate Mendelian randomization (MR) as the study’s design in the title and/or the abstract if that is a main purpose of the study | Causal relationship between diabetes mellitus and lung cancer: a two-sample Mendelian randomization and mediation analysis |
|  | **INTRODUCTION** |  |  |
| 2 | **Background** | Explain the scientific background and rationale for the reported study. What is the exposure? Is a potential causal relationship between exposure and outcome plausible? Justify why MR is a helpful method to address the study question | Diabetes mellitus (DM) is the common comorbidity with lung cancer (LC), and metabolic disorders have been identified as significant contributors to the pathogenesis of both DM and LC. The causality between diabetes mellitus and lung cancer is still controversial. Further investigation is needed to determine the causal relationships. |
| 3 | **Objectives** | State specific objectives clearly, including pre-specified causal hypotheses (if any). State that MR is a method that, under specific assumptions, intends to estimate causal effects | We aimed to investigate the causal effects of DM on the risk of LC, and the mediating role of blood metabolites in this relationship was further explored. |
|  | **METHODS** |  |  |
| 4 | **Study design and data sources** | Present key elements of the study design early in the article. Consider including a table listing sources of data for all phases of the study. For each data source contributing to the analysis, describe the following: |  |
|  | a) | Setting: Describe the study design and the underlying population, if possible. Describe the setting, locations, and relevant dates, including periods of recruitment, exposure, follow-up, and data collection, when available. | GWAS data on T2DM were derived from 159,208 participants (2,676 cases and 132,532 controls). GWAS data on T1DM were derived from 520,580 participants (18,942 cases and 501,638 controls). For LC, the GWAS data were obtained from FinnGen Consortium (412,181 participants). The genetic effect of the corresponding SNPs on serum metabolites was obtained from 8,299 individuals from the Canadian Longitudinal Study on Aging (CLSA) cohort. All individuals included in the study were of European ancestry. |
|  | b) | Participants: Give the eligibility criteria, and the sources and methods of selection of participants. Report the sample size, and whether any power or sample size calculations were carried out prior to the main analysis | The FinnGen study (https://www.finngen.fi/en) is an ongoing research project that utilizes samples from a nationwide network of Finnish biobanks and digital healthcare data from national health registers. FinnGen aims to produce genomic data with linkage to health register data of 500,000 biobank participants. |
|  | c) | Describe measurement, quality control and selection of genetic variants | Genetic instruments that met the level of p-value < 5×10−8 were selected as primary SNPs. Linkage disequilibrium (LD) was generated due to the physically closely located genetic variances in the genome that tended to be inherited together. The assumption of independence and random assignment was violated by the markers with LD. The genetic instruments were removed the LD regions and the high LD genomic loci were excluded from the selected SNPs to avoid non-random associations (r2 < 0.001, kb = 10000). The F-value statistics reflected the strength of instrumental variables and were calculated to remove the weak instrumental variables and the threshold was set at 10. Finally, we aligned the effect alleles for each SNP allele with the reference panels to ensure the accuracy and consistency of the data. |
|  | d) | For each exposure, outcome, and other relevant variables, describe methods of assessment and diagnostic criteria for diseases | The diagnostic criteria followed the ICD codes. |
|  | e) | Provide details of ethics committee approval and participant informed consent, if relevant | The Coordinating Ethics Committee of the Hospital District of Helsinki and Uusimaa (HUS) approved the FinnGen study protocol (number HUS/990/2017). |
| 5 | **Assumptions** | Explicitly state the three core IV assumptions for the main analysis (relevance, independence and exclusion restriction) as well assumptions for any additional or sensitivity analysis | 1. Exposures and instrumental variables (IVs) must be significantly related, 2. IVs should not be associated with confounding factors, 3. IVs should affect the outcome solely through exposure and not through other pathways. |
| 6 | **Statistical methods: main analysis** | Describe statistical methods and statistics used |  |
|  | a) | Describe how quantitative variables were handled in the analyses (i.e., scale, units, model) | Not applicable. |
|  | b) | Describe how genetic variants were handled in the analyses and, if applicable, how their weights were selected | The inverse variance weighted (IVW) method was employed as the principle approach. The MR Egger and weighted median were complementary calculations for MR assessment. |
|  | c) | Describe the MR estimator (e.g. two-stage least squares, Wald ratio) and related statistics. Detail the included covariates and, in case of two-sample MR, whether the same covariate set was used for adjustment in the two samples | The inverse variance weighted (IVW) method was employed as the principle approach because of the robust statistic power when SNPs were valid The MR egger method could estimate the effect for atypical SNPs; however, its statistical power was relatively weak. The weighted median method provided robust estimation when valid SNPs were more than 50%, and it was suitable for high pleiotropy |
|  | d) | Explain how missing data were addressed | In this MR analysis, the issue of missing data was not involved. |
|  | e) | If applicable, indicate how multiple testing was addressed | Not applicable. |
| 7 | **Assessment of assumptions** | Describe any methods or prior knowledge used to assess the assumptions or justify their validity | We performed the MR Egger regression to estimate the presence of horizontal pleiotropy to eliminate the IVs influencing the outcome from alternative pathways other than the exposure. |
| 8 | **Sensitivity analyses and additional analyses** | Describe any sensitivity analyses or additional analyses performed (e.g. comparison of effect estimates from different approaches, independent replication, bias analytic techniques, validation of instruments, simulations) | Cochran’s Q test was used to detect the heterogeneity. The random effect model was selected for high heterogeneity, whereas the fixed effect model was selected for low heterogeneity. We performed the MR Egger regression to estimate the presence of horizontal pleiotropy to eliminate the IVs influencing the outcome from alternative pathways other than the exposure. MR-PRESSO was conducted to exclude outliers and eliminate detected pleiotropy. |
| 9 | **Software and pre-registration** |  |  |
|  | a) | Name statistical software and package(s), including version and settings used | We conducted the sensitive analyses using TwoSampleMR (version 0.5.6) and MRPRESSO packages (version 1.0) in the R software (version 4.2.2). |
|  | b) | State whether the study protocol and details were pre-registered (as well as when and where) | Not applicable. |
|  | **RESULTS** |  |  |
| 10 | **Descriptive data** |  |  |
|  | a) | Report the numbers of individuals at each stage of included studies and reasons for exclusion. Consider use of a flow diagram | GWAS data on T2DM were derived from 159,208 participants (2,676 cases and 132,532 controls). GWAS data on T1DM were derived from 520,580 participants (18,942 cases and 501,638 controls). For LC, the GWAS data were obtained from FinnGen Consortium (412,181 participants). The genetic effect of the corresponding SNPs on serum metabolites was obtained from 8,299 individuals from the Canadian Longitudinal Study on Aging (CLSA) cohort. All individuals included in the study were of European ancestry. |
|  | b) | Report summary statistics for phenotypic exposure(s), outcome(s), and other relevant variables (e.g. means, SDs, proportions) | Summary data on exposure and outcomes are shown in Table 1. |
|  | c) | If the data sources include meta-analyses of previous studies, provide the assessments of heterogeneity across these studies | Cochran’s Q test was used to detect the heterogeneity. |
|  | d) | For two-sample MR:  i.  Provide justification of the similarity of the genetic variant-exposure associations between the exposure and outcome samples  ii.  Provide information on the number of individuals who overlap between the exposure and outcome studies | The data presented in this study were derived exclusively from European population samples. These samples were obtained from independent GWAS databases, ensuring minimal overlap and bias. |
| 11 | **Main results** |  |  |
|  | a) | Report the associations between genetic variant and exposure, and between genetic variant and outcome, preferably on an interpretable scale | Two-sample MR analyses illustrated the overall effect of T1DM on lung squamous cell carcinoma (LUSC) (OR: 1.040, 95% CI: 1.010 ~ 1.072, P = 0.009). |
|  | b) | Report MR estimates of the relationship between exposure and outcome, and the measures of uncertainty from the MR analysis, on an interpretable scale, such as odds ratio or relative risk per SD difference | The detailed results were shown in supplementary tables. |
|  | c) | If relevant, consider translating estimates of relative risk into absolute risk for a meaningful time period | Not applicable. |
|  | d) | Consider plots to visualize results (e.g. forest plot, scatterplot of associations between genetic variants and outcome versus between genetic variants and exposure) | The results were shown in Figures. |
| 12 | **Assessment of assumptions** |  |  |
|  | a) | Report the assessment of the validity of the assumptions | We selected the SNPs with a strong association with DM, and the large F statistics indicate that these analyzes will not be affected by weak instrument bias. And the selected SNPs were ensured to have no association with any confounding factors. |
|  | b) | Report any additional statistics (e.g., assessments of heterogeneity across genetic variants, such as *I^2^*, Q statistic or E-value) | We performed the MR Egger regression to estimate the presence of horizontal pleiotropy to eliminate the IVs influencing the outcome from alternative pathways other than the exposure. MR-PRESSO was conducted to exclude outliers and eliminate detected pleiotropy. The reverse MR was conducted to evaluate the existence of reverse-direction causal association. |
| 13 | **Sensitivity analyses and additional analyses** |  |  |
|  | a) | Report any sensitivity analyses to assess the robustness of the main results to violations of the assumptions | Cochran’s Q test was used to detect the heterogeneity. The random effect model was selected for high heterogeneity, whereas the fixed effect model was selected for low heterogeneity. |
|  | b) | Report results from other sensitivity analyses or additional analyses | Leave-one-out sensitivity analysis demonstrated the robustness of the MR results. |
|  | c) | Report any assessment of direction of causal relationship (e.g., bidirectional MR) | The reverse MR was conducted to evaluate the existence of reverse-direction causal association. |
|  | d) | When relevant, report and compare with estimates from non-MR analyses | Not applicable. |
|  | e) | Consider additional plots to visualize results (e.g., leave-one-out analyses) | The detailed results were shown in supplementary tables. |
|  | **DISCUSSION** |  |  |
| 14 | **Key results** | Summarize key results with reference to study objectives | In this MR study, we used a comprehensive assessment of the causal connection between DM on LC. Each SD increase in genetically predicted T1DM was associated with a 4.0% higher risk of LUSC. Furthermore, a two-step MR analysis revealed that the serum metabolites partially mediated the causal effect of T1DM and LUSC. Our findings shed light on the importance of metabolites as risk factors in understanding the relationship between T1DM and LUSC. |
| 15 | **Limitations** | Discuss limitations of the study, taking into account the validity of the IV assumptions, other sources of potential bias, and imprecision. Discuss both direction and magnitude of any potential bias and any efforts to address them | This study has certain limitations. First, our study only included the European population, which could influence the generalization of the results to other populations. Second, the TSMR research involved only the analysis of GWAS summary data, further stratification analysis including more covariates, such as age, gender, and stage, could be required. Third, the MR analysis indicated the exposure levels of the full life duration and could not reflect the accurate influence of exposure changes. |
| 16 | **Interpretation** |  |  |
|  | a) | Meaning: Give a cautious overall interpretation of results in the context of their limitations and in comparison with other studies | Our findings shed light on the importance of metabolites as risk factors in understanding the relationship between T1DM and LUSC. |
|  | b) | Mechanism: Discuss underlying biological mechanisms that could drive a potential causal relationship between the investigated exposure and the outcome, and whether the gene-environment equivalence assumption is reasonable. Use causal language carefully, clarifying that IV estimates may provide causal effects only under certain assumptions | The pathogenesis of LC has gained widespread attention and therefore, metabolic abnormalities have been emerging as a valuable research direction. The rapid growth and proliferation of tumor cells require nutrient acquisition, which has been characterized as an aberrant metabolic mechanism. Diabetes mellitus could induce the disorders of glycometabolism and lipid metabolism. Further metabolomics evidence has demonstrated the increasing glucose and decreasing lactate and phospholipid levels in patients with LC, and phospholipid composition was the important biomarkers for lung cancer. Therefore, metabolites possess considerable potential to serve as a conduit for further research on the relationship between diabetes mellitus and lung cancer. |
|  | c) | Clinical relevance: Discuss whether the results have clinical or public policy relevance, and to what extent they inform effect sizes of possible interventions | Our findings may provide new targets and biomarkers for T1DM and lung cancer. |
| 17 | **Generalizability** | Discuss the generalizability of the study results (a) to other populations, (b) across other exposure periods/timings, and (c) across other levels of exposure | To our knowledge, this is the first MR analysis to evaluate the relationship between DM and LC risk and assess the mediating effects of blood metabolites. In all, we provide evidence of the relationship between T1DM and LUSC through genetically predicted effects. T1DM could increase the risk of LUSC, and fatty acid and amino acid metabolites could exert the mediating effect. |
|  | **OTHER INFORMATION** |  |  |
| 18 | **Funding** | Describe sources of funding and the role of funders in the present study and, if applicable, sources of funding for the databases and original study or studies on which the present study is based | This study was supported by National Natural Science Fund of China.(82305344); Key Project of Henan Province Traditional Chinese Medicine Scientific Research (No.20-21ZYZD07;2023ZY1034;2022ZY1221). |
| 19 | **Data and data sharing** | Provide the data used to perform all analyses or report where and how the data can be accessed, and reference these sources in the article. Provide the statistical code needed to reproduce the results in the article, or report whether the code is publicly accessible and if so, where | Supplementary Material contains the original contributions, and additional inquiries may be contacted with the corresponding author. |
| 20 | **Conflicts of Interest** | All authors should declare all potential conflicts of interest | No conflict of interest existed. |

This checklist is copyrighted by the Equator Network under the Creative Commons Attribution 3.0 Unported (CC BY 3.0) license.

1. Skrivankova VW, Richmond RC, Woolf BAR, Yarmolinsky J, Davies NM, Swanson SA, et al. Strengthening the Reporting of Observational Studies in Epidemiology using Mendelian Randomization (STROBE-MR) Statement. JAMA. 2021;under review.

2. Skrivankova VW, Richmond RC, Woolf BAR, Davies NM, Swanson SA, VanderWeele TJ, et al. Strengthening the Reporting of Observational Studies in Epidemiology using Mendelian Randomisation (STROBE-MR): Explanation and Elaboration. BMJ. 2021;375:n2233.
